# Predicting 3D lip movement using facial sEMG: a first step towards estimating functional and aesthetic outcome of oral cancer surgery

**DOI:** 10.1007/s11517-016-1511-z

**Published:** 2016-07-01

**Authors:** Merijn Eskes, Maarten J. A. van Alphen, Ludi E. Smeele, Dieta Brandsma, Alfons J. M. Balm, Ferdinand van der Heijden

**Affiliations:** 1 Department of Head and Neck Oncology and Surgery, Netherlands Cancer Institute, Plesmanlaan 121, 1066 CX Amsterdam, The Netherlands; 2 MIRA Institute of Biomedical Engineering and Technical Medicine, University of Twente, Drienerlolaan 5, 7522 NB Enschede, The Netherlands; 3 Department of Oral and Maxillofacial Surgery, Academic Medical Center, Meibergdreef 9, 1105 AZ Amsterdam, The Netherlands; 4 Department of Neuro-Oncology, Netherlands Cancer Institute, Plesmanlaan 121, 1066 CX Amsterdam, The Netherlands; 5 Department of Neurology, Slotervaart Hospital, Louwesweg 6, 1066 EC Amsterdam, The Netherlands

**Keywords:** Oral cancer, Surface electromyography, Lips, State-space estimation, Kalman filter, Principal component analysis

## Abstract

In oral cancer, loss of function due to surgery can be unacceptable, designating the tumour as functionally inoperable. Other curative treatments can then be considered. Currently, predictions of these functional consequences are subjective and unreliable. We want to create patient-specific models to improve and objectify these predictions. A first step was taken by controlling a 3D lip model with volunteer-specific sEMG activities. We focus on the lips first, because they are essential for speech, oral food transport, and facial mimicry. Besides, they are more accessible to measurements than intraoral organs. 3D lip movement and corresponding sEMG activities are measured in five healthy volunteers, who performed 19 instructions repeatedly, to create a quantitative lip model by establishing the relationship between sEMG activities of eight facial muscles bilaterally on the input side and the corresponding 3D lip displacements on the output side. The relationship between 3D lip movement and sEMG activities was accommodated in a state-space model. A good relationship between sEMG activities and 3D lip movement was established with an average root mean square error of 2.43 mm for the first-order system and 2.46 mm for the second-order system. This information can be incorporated into biomechanical models to further personalise functional outcome assessment after treatment.

## Introduction

Oral cancer, including that of the lips, is the sixth most common cancer worldwide [15]. Surgery is still standard care [14] and can lead to deterioration of speech, swallowing and mastication with serious consequences on quality of life [6]. If surgical resection of a tumour results in an unacceptable loss of function, the tumour is designated as functionally inoperable, and other curative treatment options such as chemoradiotherapy or radiotherapy can serve as alternative treatments [7].

Accurate prediction of the functional consequences of surgery is an urgent need to make the right choice of treatment [19]. Functional prediction using virtual surgery is complex and involves several aspects of patient-specific anatomical geometry, biomechanical tissue properties, branching and distribution pattern of the nervous system and the muscle activation signals that control a particular function. Biomechanical modelling, including the muscular system, in the oral and oropharyngeal region, has been the subject of ongoing research [10, 16, 18, 23].

This paper focuses on the lips, since these are essential for speech, oral food transport and facial mimicry. To create a predictive model, a continuum of 3D lip shapes is needed ultimately to perform virtual surgery on a model. Former research on lip modelling, utilising surface electromyography (sEMG) of facial muscles, is more phonetic in nature and is mainly focused on the categorisation of facial expressions [4], categorisation of vowels [1] and words [2].

To our knowledge, only two studies have described lip shape modelling in combination with quantitative lip pose estimation using facial electromyography (EMG). Honda et al. [5] recorded lip motion in the 2D frontal projection of the face and sEMG signals from only one side. They used a direct linear mapping of EMG to the lip coordinates based on multiple regression analysis. A visual comparison between the measured and modelled lip shapes was made. Lucero and Munhall acquired intramuscular EMG data, using hooked-wire bipolar electrodes, of one side of the face, and simultaneously measured lip and face displacements on the other side [9]. The relationship between EMG activity and marker displacements was based on a facial finite element model and the connection between EMG feature and the steady-state force generated by the corresponding muscle was presumed to be linear. The quantitative evaluation was expressed in terms of cross-correlation between model-predicted and measured displacements of the individual markers. For markers on the lips, these cross-correlations were rather low (mean values: 0.0–0.91) with very low cross-correlation for protrusion. The instructions in these studies differed, Honda et al. used five Japanese vowels, and the subject in the study of Lucero and Munhall was asked to produce an English sentence. Both models were tested on one volunteer.

The goal of this study is twofold. First we want to demonstrate that sEMG signals contain enough information for controlling 3D dynamic models of facial expressions, particularly lip movements. The second goal is to establish the optimal processing configuration to extract information from facial sEMG data. To avoid the complexity and pitfalls of detailed biomechanical models, we first focus on an empirical model. If the results of this empirical model are promising, the premise is justified that sEMG signals are very useful to solve the ambiguity problems in inverse dynamic modelling [17]. In addition, our study also should reveal which sEMG processing configuration, e.g. sEMG feature type and time window, is most promising for sEMG-based inverse modelling. The ambiguity problem of inverse dynamic modelling stems from the fact that a desired movement can be accomplished in various ways [17]. The activation pattern that causes the desired movement is not unique. The addition of the sEMG could provide further information about a patient-specific activation pattern.

## Methods

### Volunteers and data acquisition

**Fig. 1:**
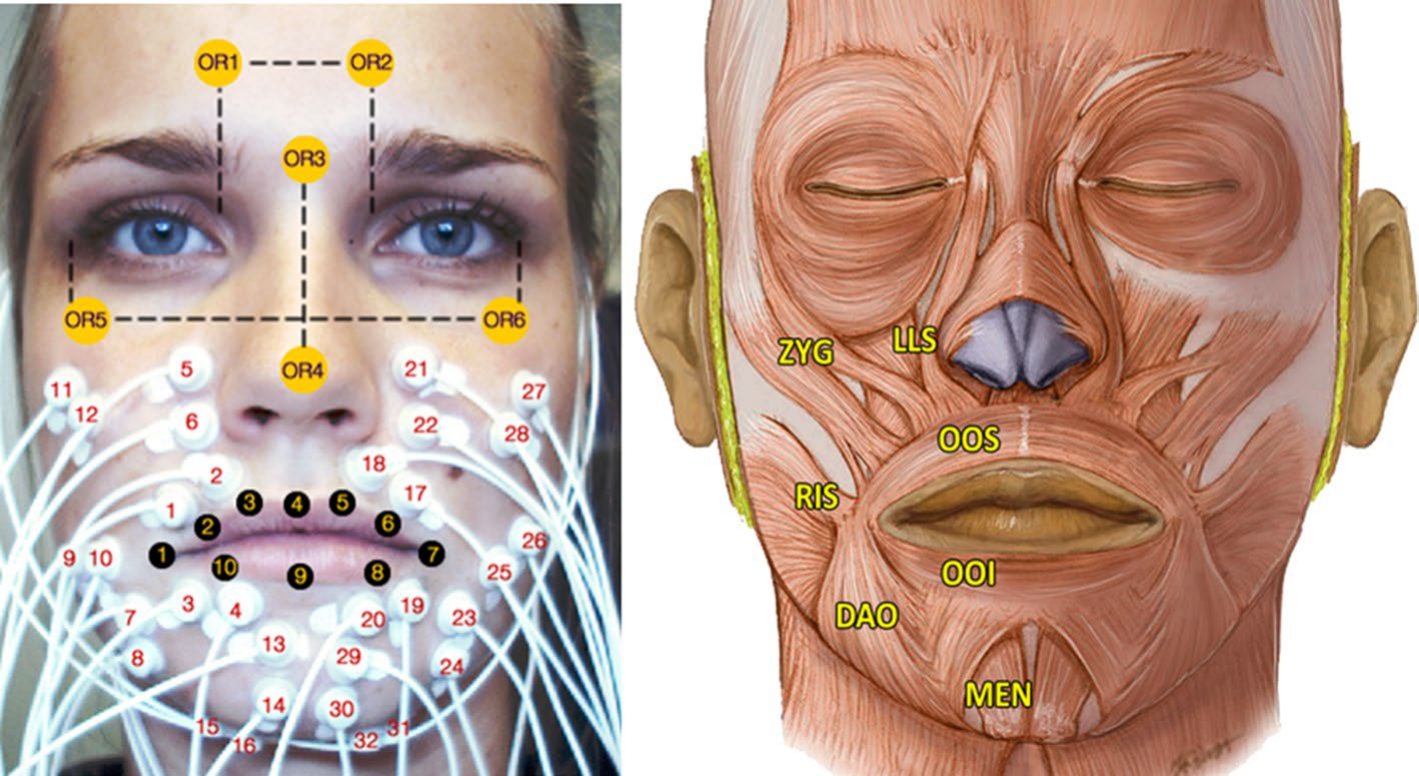
*Left* locations of electrodes, orientation markers and lip markers. *Right* measured facial muscles (excluding the digastric muscle) [16] © Springer, with permission of Springer

Data were obtained from five healthy volunteers $(k = 1, \ldots ,5)$ consisting of two males and three females, age ranging from 21 to 30. The recording sites of the skin were cleansed with NuPrep abrasive gel and alcohol. The sEMG signals were recorded using a TMSi^®^ Porti™ system (TMSi^®^, Oldenzaal, the Netherlands). The micro-sEMG sintered disc-shaped surface electrodes (1.5 mm diameter, Ag/AgCL, with shielded cables) were placed above eight muscles on both sides of the face $(n = 1, \ldots ,16)$, as shown in Fig. 1. The locations were chosen based on human lip anatomy and a study of Lapatki et al. [8] showing the effects on lip shapes. Additionally, a common ground reference electrode was applied with a self-adhesive button electrode on the left wrist. In Table 1, the measured muscles, their functions and electrode number, corresponding to the numbering in Fig. 1, are given. Sixteen facial markers were defined using a skin marker. Ten markers covered the lip contour $(m = 1, \ldots ,10)$. The other six markers $(m_{\text{OR}} = 1, \ldots ,6)$ were located on the face (cheeks, nose and forehead; see Fig. 1) and were used to compensate for head movement. The volunteers were positioned in front of a triple-camera set-up consisting of three cameras (Basler avA1000-100gc), which recorded the lip movement at 100 frames per second.

**Table 1:** Muscle, muscle function and corresponding electrode number

Muscle	Function	Electrodes number (right/left)
Zygomaticus major (ZYG)	Elevates the corners of the mouth in lateral direction	11, 12/27,28
Risorius (RIS)	Retracts angle of mouth	9, 10/25, 26
Orbicularis oris superior (OOS)	Narrows orifice of mouth, purses lips and puckers lip edges	1, 2/17, 18
Orbicularis oris inferior (OOI)	Narrows orifice of mouth, purses lips and puckers lip edges	3, 4/19, 20
Mentalis (MEN)	Draws up the skin of the chin and causes the lower lip to protrude	13, 14/29, 30
Depressor anguli oris (DAO)	Draws the corners of the mouth downwards and laterally	7, 8/23, 24
Levator labii superioris (LLS)	Elevates and everts upper lip	5, 6/21, 22
Digastricus (DIG)	Depresses mandible, opening mouth and/or elevates larynx	15, 16/31, 32

### Instructions to volunteers

A study of van Son et al., showed that Dutch (experienced) lip readers were able to recognise five consonantal and five vowel visemes [21]. Visemes are groups of speech sounds that are visually indistinguishable. These Dutch viseme instructions were used in this study. Besides these visemes, six facial expressions that maximised independent contraction of the measured muscles were included. These selected expressions were based on the work of Lapatki et al. [8]. Lastly, two asymmetric motions were performed from left to right to left with closed lips, and with open lips, and one dynamic motion transfer between two expressions; purse lips to voluntary smiling to purse lips. Each volunteer was asked to repeat the 19 instructions $(i = 1, \ldots ,19)$ five times $(r = 1, \ldots ,5)$. The instructions are shown Table 2.

**Table 2:**
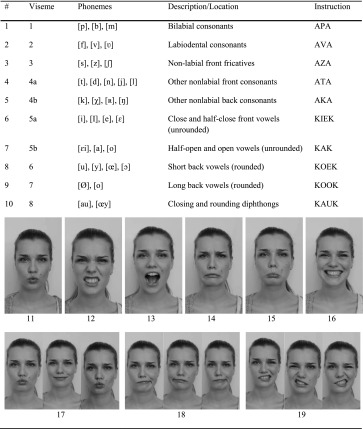
Instructions: visemes (1–10), facial expressions (11–17) and asymmetric movements (18–19)

### Data processing and analysis

#### sEMG preprocessing

The sEMG signals $s_{n} (t,i,r)$ were recorded in bipolar configuration with a sample frequency of 2048 Hz. Here, $t$ is the time index, $i$ is the instruction and $r$ is the repetition number. All recorded signals were band-pass filtered with a high- and low-pass fourth-order Butterworth filter with cut-off frequencies of, respectively, 15 and 500 Hz, in accordance with van Boxtel [20].

Many different sEMG feature types have been proposed in the literature. Based on the results of Phinyomark et al., who examined 37 feature types, and our results of a preliminary experiment, we chose to investigate four feature types given in Table 3 [11]. Thresholds for the WAMP feature $(x_{\lim } )$ were set to 10 and 20 mV. With all 16 sEMG channels stacked in a vector the result is denoted: ${\mathbf{g}}_{f} (t,i,r) \in {\mathbf{\mathbb{R}}}^{16}$, with $f = 1, \ldots ,5$ the feature type. Features were calculated over a sliding window with maximum overlap. The different window lengths examined were: 50, 100, 150, 200, 250 and 300 ms.

**Table 3:** sEMG features

$f$	Feature	Formula
1	RMS	$\sqrt {\frac{1}{N}\mathop \sum \nolimits_{i = 1}^{N} x_{i}^{2} }$
2	MAV	$\frac{1}{N}\mathop \sum \nolimits_{i = 1}^{N} \left| {x_{i} } \right|$
3	WL	$\mathop \sum \nolimits_{i = 1}^{N - 1} \left| {x_{i + 1} - x_{i} } \right|$
4 $x_{\lim } = 10{\text{ mV}}$	WAMP	$\mathop \sum \nolimits_{i = 1}^{N - 1} \left[ {f\left( {\left| {x_{n} - x_{n + 1} } \right|} \right)} \right]$
5 $x_{\lim } = 20\;{\text{mV}}$		with $$ f\left( x \right) = \left\{ {\begin{array}{*{20}c} 1 & {{\text{if }}x \ge x_{\lim } } \\ 0 & {\text{otherwise}} \\ \end{array} } \right. $$

The videos were recorded concurrently with the sEMG. To synchronise the recorded sEMG signals with the video recordings a synchronisation pulse was fed to the TMSi^®^ Porti™ system when the cameras started their recordings. Thereafter the sEMG signals were cut and resampled to 100 Hz, equivalent to the frame rate of the cameras.

There is a small time delay between a sEMG activity and the corresponding muscle activation. It is difficult to define a default value for this delay. Honda et al. [5] used 70 ms, whereas Vatikiotis et al. [22] used different delays varying from 0 to 100 ms. By minimising the estimation errors of the lip marker positions, we empirically determined a mean muscle activation delay of 30 ms, which we compensated in all records.

#### Video preprocessing

The facial markers were tracked in the images of the three cameras, and the 2D coordinates were reconstructed to a set of 3D coordinates. The root mean square (RMS) error of the 3D localisation of markers, obtained via the leave-one-out method, was 0.73 mm. The resulting 3D positions of the ten markers on the lip, corrected for head movement, are denoted by ${\mathbf{X}}(t,i,r) \in {\mathbb{R}}^{30}$.

#### The measurement model

State-space estimation requires the availability of a measurement model that links the sEMG features $$ {\mathbf{g}}_{f} (t,i,r) $$ to marker positions $$ {\mathbf{X}}(t,i,r) $$. The relationship between these quantities is nonlinear, whereas a linear model was preferred. To arrive at a linear approximation, a truncated Taylor series in $$ {\mathbf{g}}_{f} (t,i,r) $$ up to order two was used. For this, the 16D feature vector $$ {\mathbf{g}}_{f} (t,i,r) $$ was augmented with all the 136 quadratic terms and cross products of its elements yielding a $$ 152 \times D $$ vector $$ {\mathbf{\underset{\raise0.3em\hbox{$\smash{\scriptscriptstyle-}$}}{g} }}_{f} (t,i,r) $$.

To establish the measurement model, first a principal component analysis (PCA) was applied. Suppose that a training set consisting of $$ J $$ observed sEMG features $$ {\mathbf{\underset{\raise0.3em\hbox{$\smash{\scriptscriptstyle-}$}}{g} }}(j) $$ and corresponding marker positions $$ {\mathbf{X}}(j) $$, with $$ j = 1, \ldots ,J $$, is available. The exact construction of this training set will be explained later. PCA was applied to the concatenation of these vectors:

1 $$ {\mathbf{z}}(j)\mathop = \limits^{\text{def}} \left[ {\begin{array}{*{20}c} {{\mathbf{X}}(j)} \\ {{\mathbf{\underset{\raise0.3em\hbox{$\smash{\scriptscriptstyle-}$}}{g} }}(j)} \\ \end{array} } \right] $$

The dimension of the vectors ${\mathbf{z}}(j)$ is 182. The set was normalised with respect to mean and variance of each element before applying the PCA, because the sEMG features and the 3D coordinates present two different physical dimensions. The PCA resulted in a $182 \times D$-dimensional orthogonal matrix ${\mathbf{Y}}$ containing the first $D$ principal components of the set. Encoding of a vector ${\mathbf{z}}$ in a $D$-dimensional coefficient vector **b**, and subsequent decoding, occurs according to:

2 $$ \begin{array} {l} {\mathbf{b}} = {\mathbf{Y}}^{\text{T}} {\mathbf{z}}\,\left( {\text{encoding}} \right) \hfill \\ {\hat{\mathbf{z}}} = {\mathbf{Yb}}\,\left( {\text{decoding}} \right) \hfill \\ \end{array} $$

The mean of the coefficient vector ${\mathbf{b}}$ is zero, and the covariance matrix ${\mathbf{C}}_{{\mathbf{b}}}$ is diagonal with the elements sorted in descending order.

To arrive at a (pseudo-) linear measurement model, we constructed the matrix ${\mathbf{Y}}_{{\mathbf{g}}}$ from ${\mathbf{Y}}$ by leaving out the first 30 rows corresponding to the positions ${\mathbf{X}}$. We then have:

3 $$ {\hat{\mathbf{\underset{\raise0.3em\hbox{$\smash{\scriptscriptstyle-}$}}{g} }}} = {\mathbf{Y}}_{{\mathbf{g}}} {\mathbf{b}} $$

Suppose that the residuals of $$ {\hat{\mathbf{\underset{\raise0.3em\hbox{$\smash{\scriptscriptstyle-}$}}{g} }}} $$ are given by $$ {\mathbf{v}} $$, such that $$ {\mathbf{\underset{\raise0.3em\hbox{$\smash{\scriptscriptstyle-}$}}{g} }} = {\hat{\mathbf{\underset{\raise0.3em\hbox{$\smash{\scriptscriptstyle-}$}}{g} }}} + {\mathbf{v}} $$, then:

4 $$ {\mathbf{\underset{\raise0.3em\hbox{$\smash{\scriptscriptstyle-}$}}{g} }} = {\mathbf{Y}}_{{\mathbf{g}}} {\mathbf{b}} + {\mathbf{v}} $$

This can be regarded as a linear measurement model of $$ {\mathbf{b}} $$ with $$ {\mathbf{\underset{\raise0.3em\hbox{$\smash{\scriptscriptstyle-}$}}{g} }} $$ the measurement vector, $$ {\mathbf{Y}}_{{\mathbf{g}}} $$ the measurement matrix and $$ {\mathbf{v}} $$ the measurement noise. The covariance matrix $$ {\mathbf{C}}_{{\mathbf{v}}} $$ of $$ {\mathbf{v}} $$ is a $$ 152 \times 152 $$-dimensional matrix which can easily be estimated from the training set. Due to augmentation of $$ {\mathbf{g}} $$ with quadratic terms, the measurement noise is not guaranteed to be uncorrelated, and the matrix $$ {\mathbf{C}}_{{\mathbf{v}}} $$ might be non-diagonal.

#### State-space modelling

For dynamic modelling, two state-space models were implemented, a first-order and a second-order system. In the first-order system, a time series ${\mathbf{b}}(t)$ was modelled dynamically with:

5 $$ {\mathbf{b}}(t + 1) = {\mathbf{Fb}}(t) + {\mathbf{w}}(t) $$

${\mathbf{b}}(t)$ is the $D$-dimensional state vector, and ${\mathbf{F}}$ is the $D \times D$ system matrix. The process noise ${\mathbf{w}}(t)$ was assumed to be zero mean and uncorrelated in time. Its covariance matrix is ${\mathbf{C}}_{{\mathbf{w}}}$. The system matrix ${\mathbf{F}}$ was estimated from the training set using ${\text{E}}\left[ {{\mathbf{b}}(t + 1){\mathbf{b}}^{T} (t)} \right] = {\mathbf{F}}\;{\text{E}}\left[ {{\mathbf{b}}(t){\mathbf{b}}^{T} (t)} \right]$. Here, ${\text{E}}\left[ {} \right]$ is the expectation operator; hence, ${\hat{\mathbf{F}}} = \overline{{{\mathbf{b}}(t + 1){\mathbf{b}}^{T} (t)}} \left( {\overline{{{\mathbf{b}}(t){\mathbf{b}}^{T} (t)}} } \right)^{ - 1}$. The covariance matrix ${\mathbf{C}}_{{\mathbf{w}}}$ can be estimated from the training set using ${\mathbf{w}}(t) = {\mathbf{b}}(t + 1) - {\mathbf{Fb}}(t)$. Preliminary experiments showed that both ${\mathbf{F}}$ and ${\mathbf{C}}_{{\mathbf{w}}}$ are diagonal. This was expected as the PCA decorrelated the coefficients ${\mathbf{b}}(t)$. In addition, the system matrix ${\mathbf{F}}$ appeared to approximate the identity matrix ${\mathbf{I}}$. This was also expected as the sampling period, 10 ms, is rather small compared to the expected time constant of lip motions.

In the second-order system, the state vector was defined as:

6 $$ {\mathbf{x}}(t)\mathop = \limits^{\text{def}} \left[ {\begin{array}{*{20}c} {{\mathbf{b}}(t - 1)} \\ {{\mathbf{b}}(t)} \\ \end{array} } \right] $$

with associated state equation:

7 $$ {\mathbf{x}}(t + 1) = {\mathbf{Fx}}(t) + {\mathbf{w}}(t)\quad {\text{with}}\quad {\mathbf{F}} = \left[ {\begin{array}{*{20}c} {\mathbf{0}} & \quad {\mathbf{I}} \\ {{\mathbf{F}}_{1} } & \quad {{\mathbf{F}}_{2} } \\ \end{array} } \right] $$

Preliminary experiments showed that the submatrices ${\mathbf{F}}_{1}$ and ${\mathbf{F}}_{2}$ are diagonal which again is in line with the uncorrelatedness of the coefficients ${\mathbf{b}}(t)$. Equation (7) models $D$ decoupled second-order autoregressive (AR) models, one for each coefficient $b_{n} (t)$ in ${\mathbf{b}}(t)$, i.e.

8 $$ b_{n} (t + 1) = \alpha_{n} b_{n} (t) + \beta_{n} b_{n} (t - 1) + w_{n} (t)\quad {\text{with}}\quad n = 1, \ldots ,D $$

where $\alpha_{n}$ is a diagonal element from ${\mathbf{F}}_{2}$ and $\beta_{n}$ a diagonal element from ${\mathbf{F}}_{1}$. The AR models represent second-order differential equations in the continuous time that are characterised by their natural frequencies $f_{n}$ and relative damping $\zeta_{n}$ given by:

9 $$ f_{n} = \frac{{\sqrt {1 - \alpha_{n} - \beta_{n} } }}{2\pi T}\quad {\text{and}}\quad \zeta_{n} = \frac{{ - \alpha_{n} - 2}}{{2\sqrt {1 - \alpha_{n} - \beta_{n} } }} $$

where $T$ is the sampling period. The natural frequency determines the bandwidth of the corresponding coefficient. The damping determines the spectrum of the signal around the natural frequency. We used these parameters to fine-tune the state-space model during training.

The process noise ${\mathbf{w}}(t)$ has zero elements in the first $D$ elements. Thus, the covariance matrix is built as follows:

10 $$ {\mathbf{C}}_{{\mathbf{w}}} = \left[ {\begin{array}{*{20}c} {\mathbf{0}} & \quad {\mathbf{0}} \\ {\mathbf{0}} & \quad {{\mathbf{C}}_{22} } \\ \end{array} } \right] $$

${\mathbf{C}}_{22}$ is a diagonal matrix as the coefficients of a PCA are uncorrelated. Preliminary results showed that this was indeed the case. To determine the influence of dynamic modelling we also performed static modelling by enforcing the Kalman filter, which is described below, to use only measurements, and to ignore the predictions. This was effectuated by setting the standard deviation of the process noise to almost infinity.

#### Estimation

The estimation of the coefficients of the PCA was done with a discrete Kalman filter. The dimension of the state vector is in the first-order system $$ D $$ and in the second-order system $$ 2 \times D $$. In practice, $$ D $$, being the result of the PCA, is much smaller than the dimension of the measurement vector, $$ {\mathbf{\underset{\raise0.3em\hbox{$\smash{\scriptscriptstyle-}$}}{g} }}(t) $$, which is 152. Therefore, the Kalman filter was used in the following form:

11 $$ \begin{aligned} &\left. {\begin{array}{*{20}l} {{\hat{\mathbf{x}}}(t|t - 1) = {\mathbf{F}}\hat{\mathbf{x}}(t - 1|t - 1)} \hfill \\ {{\mathbf{C}}(t|t - 1) = {\mathbf{FC}}(t - 1|t - 1){\mathbf{F}}^{T} + {\mathbf{C}}_{w} } \hfill \\ \end{array} } \right\}{\text{prediction}} \hfill \\ &\left. {\begin{array}{*{20}l} {{\mathbf{C}}(t|t) = \left( {{\mathbf{C}}^{ - 1} (t|t - 1) + {\mathbf{H}}^{T} {\mathbf{C}}_{{\mathbf{v}}}^{ - 1} {\mathbf{H}}} \right)^{ - 1} } \hfill \\ {{\hat{\mathbf{x}}}(t|t) = {\mathbf{C}}(t|t)\left( {{\mathbf{C}}^{ - 1} (t|t - 1){\hat{\mathbf{x}}}(t|t - 1) + {\mathbf{H}}^{T} {\mathbf{C}}_{{\mathbf{v}}}^{ - 1} {\mathbf{\underset{\raise0.3em\hbox{$\smash{\scriptscriptstyle-}$}}{g} }}(t)} \right)} \hfill \\ \end{array} } \right\}{\text{updating}} \hfill \\ \end{aligned} $$

This is computationally more efficient than the typical form. In Eq. (11), ${\mathbf{H}}$ is the measurement matrix, which equals ${\mathbf{Y}}_{{\mathbf{g}}}$ in the first-order system and $$ \left[ {\begin{array}{*{20}c} {\mathbf{0}} & {{\mathbf{Y}}_{{\mathbf{g}}} } \\ \end{array} } \right] $$ in the second-order system.

#### Training and testing

The algorithm needs training data to find the PCA components ${\mathbf{Y}}$, the covariance matrices ${\mathbf{C}}_{{\mathbf{v}}}$ and ${\mathbf{C}}_{{\mathbf{w}}}$ and in case of the first-order system the system matrix ${\mathbf{F}}$ and in case of the second-order system, its submatrices ${\mathbf{F}}_{1}$ and ${\mathbf{F}}_{2}$. The dimension $D$ of the PCA is a design parameter. Additional design parameters were introduced to fine-tune the models. These were as follows:

- The measurement noise covariance matrix ${\mathbf{C}}_{{\mathbf{v}}}$ was corrected with a regularisation parameter $c_{v}$. Instead of ${\mathbf{C}}_{{\mathbf{v}}}$, the matrix $(1 - c_{v} ){\mathbf{C}}_{{\mathbf{v}}} + c_{v} \overline{{{\mathbf{C}}_{{\mathbf{v}}} (\ell ,\ell )}} \;{\mathbf{I}}$, with $\overline{{{\mathbf{C}}_{{\mathbf{v}}} (\ell ,\ell )}} \;$ the average of the diagonal elements, was used.
- The process noise covariance matrix ${\mathbf{C}}_{{\mathbf{w}}}$ was corrected with a regularisation parameter $c_{w}$. That is, the submatrix ${\mathbf{C}}_{22}$ was replaced by the matrix $(1 - c_{w} ){\mathbf{C}}_{22} + c_{w} {\text{diag}}(\overline{{{\mathbf{C}}_{22} (\ell ,\ell )}} )$. Here, ${\text{diag}}(\overline{{{\mathbf{C}}_{22} (\ell ,\ell )}} )$ is the diagonal matrix that is built with a smoothed version of the diagonal elements of ${\mathbf{C}}_{22}$.
- The diagonal matrices ${\mathbf{F}}_{1}$ and ${\mathbf{F}}_{2}$, which holds the second-order AR parameters $\alpha_{n}$ and $\beta_{n}$, respectively, were corrected by application of a proportionality constant to the corresponding natural frequencies and damping by constants $c_{f}$ and $c_{d}$. So, instead of $f_{n}$ and $\zeta_{n}$, the parameters $c_{f} f_{n}$ and $c_{d} \zeta_{n}$ were used.

This resulted into three design parameters, $D$, $c_{v}$ and $c_{w}$, for the first-order system and five design parameters, $D$, $c_{v}$, $c_{w}$, $c_{f}$ and $c_{d}$, for the second-order system. These parameters were optimised using training data.

**Fig. 2:**
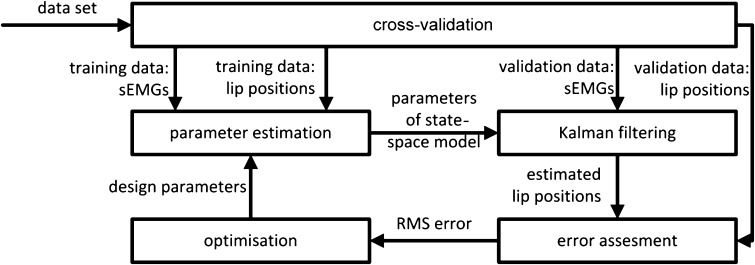
Optimisation and evaluation of design parameters using cross-validation

We performed cross-validation for training and testing. The procedure is depicted in Fig. 2. It was applied per volunteer, per feature type and per window size. Data from the various instructions were pooled by concatenating the data: $$ {\mathbf{\underset{\raise0.3em\hbox{$\smash{\scriptscriptstyle-}$}}{g} }}_{f} (t,r) = \left[ {\begin{array}{*{20}c} {{\mathbf{\underset{\raise0.3em\hbox{$\smash{\scriptscriptstyle-}$}}{g} }}_{f} (t,1,r)} & \cdots & {{\mathbf{\underset{\raise0.3em\hbox{$\smash{\scriptscriptstyle-}$}}{g} }}_{f} (t,19,r)} \\ \end{array} } \right] $$. The data from four repetitions were pooled to get the training data: $$ {\mathbf{\underset{\raise0.3em\hbox{$\smash{\scriptscriptstyle-}$}}{g} }}_{f} (t) = \left[ {\begin{array}{*{20}c} {{\mathbf{\underset{\raise0.3em\hbox{$\smash{\scriptscriptstyle-}$}}{g} }}_{f} (t,1)} & \cdots & {{\mathbf{\underset{\raise0.3em\hbox{$\smash{\scriptscriptstyle-}$}}{g} }}_{f} (t,4)} \\ \end{array} } \right] $$. Testing was performed on the fifth repetition. Cross-validation took place by rotating the repetitions. The final evaluation criterion was defined as the RMS of the error calculated over all marker coordinates and all repetitions. The design parameters were obtained by minimisation of the RMS error by varying these parameters one by one and applying successive parabolic optimisation. The one-sided paired Wilcoxon test was used to test for significant differences between the static and the two dynamic systems. The one-sided test was justified because the static model is in fact included in the dynamic model as a special case, and as such the optimised performance of the dynamic model cannot be less than the static model.

## Results

The best results for the static, first-order and second-order state-space models are summarised in Table 4 for the individual subjects and on average. The first-order system for state-space modelling performed best on average, with a RMS error of 2.43 mm on average. The first-order and the second-order system showed statistically significant better results than the static system (*p* = 0.03). No significant difference was found between the two dynamic systems. Four subjects showed the best results when the WAMP feature was used. The optimal threshold $x_{\lim }$ differed between the subjects. A window length of 200 ms performed best on average. The average RMS error was 2.46 mm for the second-order state-space model, also using the WAMP feature with $x_{\lim } = 10\;{\text{mV}}$ and a window length of 200 ms. As expected, static modelling showed poorer results, but performed also best when the WAMP feature was used.

**Table 4:** RMS error, optimal feature and window, found per volunteer and on average for the static, first-order and second-order dynamic model

Volunteer	1	2	3	4	5	Average
Static						
RMS error (SD) (mm)	2.34 (0.21)	2.55 (0.21)	3.02 (0.12)	2.32 (0.15)	2.92 (0.14)	2.70 (0.19)
Feature	WAMP (20 mV)	WAMP (20 mV)	WAMP (20 mV)	WAMP (10 mV)	WAMP (10 mV)	WAMP (10 mV)
Window (ms)	200	200	300	250	300	250
First-order system						
RMS error (SD) (mm)	2.10 (0.17)	2.29 (0.19)	2.64 (0.17)	2.10 (0.19)	2.66 (0.19)	2.43 (0.18)
Feature	WAMP (20 mV)	WL	WAMP (20 mV)	WAMP (10 mV)	WAMP (10 mV)	WAMP (10 mV)
Window (ms)	200	200	200	250	250	200
Second-order system						
RMS error (SD) (mm)	2.02 (0.19)	2.42 (0.18)	2.58 (0.18)	2.13 (0.21)	2.66 (0.21)	2.46 (0.18)
Feature	WAMP (20 mV)	WL	WAMP (20 mV)	WAMP (10 mV)	WAMP (10 mV)	WAMP (10 mV)
Window (ms)	200	250	150	200	200	200

The RMS errors for the different features for the first-order and second-order system are presented in Table 5. It can be seen that for both $x_{\lim } = 10\;{\text{mV}}$ and $x_{\lim } = 20\; {\text{mV}}$ the WAMP feature performance was comparable. Regarding the other features, the WL performed slightly worse compared to the WAMP. RMS and MAV showed the poorest results.

**Table 5:** Optimal settings averaged over the volunteers obtained per feature and system order

	RMS		MAV		WL		WAMP10		WAMP20	
System order	First	Second	First	Second	First	Second	First	Second	First	Second
RMS error (mm)	2.74	2.70	2.67	2.64	2.50	2.50	2.43	2.46	2.45	2.46
Window (ms)	200	200	250	200	200	200	200	200	200	200

**Fig. 3:**
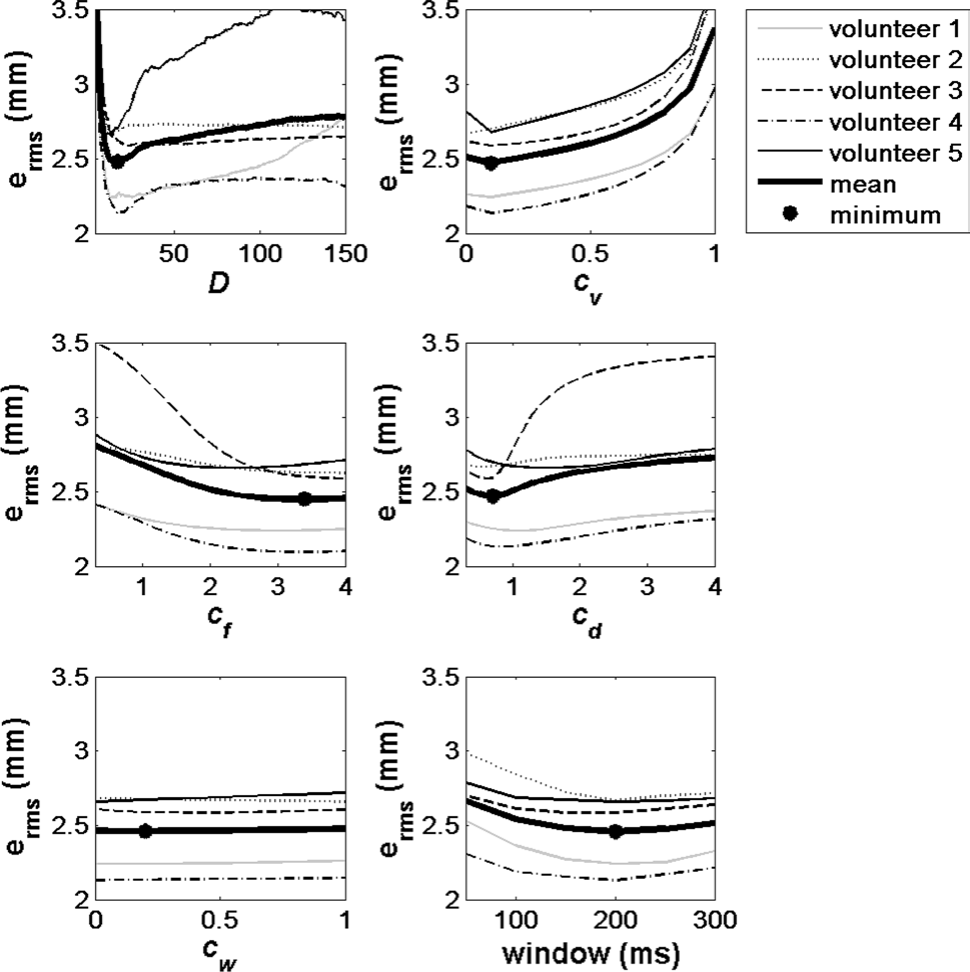
Dependence of the RMS error on the various parameters and window length in the second-order system

The influence of the different parameters and window length on the error in the second-order system can be seen in Fig. 3. Each graph shows the influence of one parameter on the RMS error while the others are set to values which lead to the optimal results on average. The dimension of the PCA, $D$, shows a plateau after 20 components. Optimal values of 0.1 and 3.4 were found for $c_{v}$ and $c_{f}$, respectively. The regularisation parameter $c_{d}$ had the minimum error at a factor of 0.7. The constant $c_{w}$ showed little influence but had an optimum on average at 0.2. The window lengths showed a similar trend in all subjects, with the best results for medium length windows. For the first-order system, comparable values were found.

## Discussion

For the prediction of the functional and aesthetic consequences of treatment in oral cancer, dynamic models of the lips are required. Biomechanical modelling is physics based and as such the most direct method to predict these consequences. However, finding the patient-specific muscle activation signals needed for the biomechanical models is difficult [24]. sEMG signals may contain information to help in finding these patient-specific activations signals. To find the optimal sEMG processing configuration and to prove that sEMG signals contain sufficient information to do so, the current study describes an empirically derived model that is able to estimate the dynamics of lip displacements with an average RMS error of 2.43 mm. This empirical model is sEMG driven, which incorporates volunteer-specific information. As far as we know, we are the first who expressed distance errors of lip motion prediction based on sEMG features.

The approach used here, incorporated the dynamics of the system by means of a state-space model. To test whether dynamical modelling was superior to static modelling, we implemented both. As expected, incorporation of dynamics improved the model. In comparison with the static system RMS errors decreased in every volunteer, with an average of 0.27 mm for the first-order system and 0.24 mm for the second-order system. An advantage of a dynamic system is that bandwidth can be sacrificed to improve the signal-to-noise ratio. Apparently, in the current application such a sacrifice pays off, but not drastically.

The difference between the first-order and second-order system is negligible. A higher-order system has more parameters which have to be estimated, making the filter more sensitive for deviations in those parameters to the optimum settings. An optimal equilibrium has to be found between modelling accurate dynamic behaviour for which higher-order systems are beneficial, and confining the impact of errors in the estimated parameters for which a lower-order system is preferred. In this study, the advantages of a second-order system over a first-order system did not outweigh the errors induced by the deviations in the estimated parameters.

The fudging parameters were used to optimise the model per volunteer and hence make it volunteer-specific. Only the regularisation parameter $c_{w}$ for the process noise covariance matrix ${\mathbf{C}}_{{\mathbf{w}}}$ did not have much influence. For each parameter, a similar trend was seen regarding the optimal values, but the level of influence differed per volunteer. The optimal values found in this study can be used to set the limits for future volunteers, thereby decreasing computational time of the parabolic optimisation. The dimension of the PCA reached a plateau at 20, four dimensions more than the original 16 dimensions, suggesting that the cross products of the sEMG provided additional information. Preliminary experiments indicated that leaving out all the nonlinear cross products seriously deteriorated the results. Hence, the nonlinearity of the system is substantial. Finally, window length was optimal at medium lengths. A possible explanation is that short window lengths are prone to noise, whereas longer window lengths smooth the signals too much.

The different features also had a noticeable influence on the RMS error. The WAMP features with $x_{\lim } = 10$ and $x_{\lim } = 20\;{\text{mV}}$ were most promising. Perhaps that thresholds in-between these values could perform better. One can also think of optimising $x_{\lim }$ per muscle channel for optimal results. The widely used RMS feature performed worse. This was also found by Phinyomark et al. [11].

Because of different error assessments, the differences of our model compared to studies in the literature will be discussed qualitatively. The main differences are stated in Table 6. The current model showed results for a more extensive set of instructions, including asymmetric movements. Furthermore, more 3D lip markers and more muscles were included. Our model predicts 3D movement of the lips based on measurements on both sides of the face and therefore is more realistic. Honda et al. used a linear statistical approach, which is inadequate for modelling nonlinear soft tissue changes. To allow the model to cope with nonlinear behaviour, we calculated the cross products of the sEMG signal features to add nonlinearity. The model of Honda et al. did not include the factor of jaw movement, making the estimation of vertical movement prone to errors. Lucero and Munhall controlled jaw movement by tracking an optical marker instead of EMG signals. Non-surprisingly, the modelled facial tissue followed this movement well. We added sEMG measurements of the digastric muscle to make the prediction of jaw movement possible. Both Honda et al. and Lucero and Munhall measured EMG signals on one side of the face, disregarding asymmetry in facial morphology and lip movement as described by Campbell [3]. The use of hooked-wire, as used by Lucero and Munhall, or needle electrodes is attractive to overcome the problem of crosstalk, but for clinical applications this will be impractical because they are invasive and consequently patient-unfriendly. Therefore, in generating future personalised 3D models controlled by EMG signals, sEMG will have our preference, being easy applicable and patient-friendly. Furthermore, we tested our model in five subjects, indicating it is general applicable.

**Table 6:** Differences in experimental set-up in related studies

	Current study	Honda et al. [5]	Lucero and Munhall [9]
Dimension	3D	2D	3D
Measurement	Bilateral	Unilateral	Unilateral
Muscles	16	6	7
Lip markers	10	7	5
EMG	Surface	Surface	Hooked-wire
Jaw movement	Digastric muscle	Omitted	Manual
Model	PCA MMSE + Kalman	Multiple regression	FEM
Instructions	Visemes and facial expressions (19)	Japanese vowels (5)	English sentence
Volunteers	5	1	1
Error assessment	RMS distance	Visual	Visual and cross-correlations

A limitation of the proposed set-up is the high number of required sEMG electrodes. This makes the current approach time-consuming which is inconvenient in future clinical practice. Monopolar derivations can be used to halve the number of electrodes; however, this configuration does not reduce unwanted noise from the recordings by using the differential amplifier design [12]. Another approach to lessen the number of electrodes is to identify less influential muscle channels for the estimation of motion, and include only those channels that affect motion prediction most.

Another difficulty is the variability in muscle anatomy, and overlying soft tissue, which makes standardisation of the measurements difficult. Additionally, physiological orofacial functions usually require simultaneous contraction of various muscles. These muscles therefore lack training in isolated contractions, resulting in relatively high co-contraction of muscles. Also volunteers can use different muscle activation patterns to perform the same instruction. We saw similar results when facial expressions were performed as described by Schumann et al. [13]. Most volunteers were able to selectively activate the LLS, whereas most volunteers had difficulty in pulling their lip corners down (DAO). Purse lips, pout lips and voluntary smiling all induced multiple muscle activations, showing the difficulty in selective muscle activation of facial muscles.

The two main pillars of our study were to demonstrate that sEMG signals contain sufficient information to control 3D dynamic models of lip movements and to determine the best sEMG processing configuration for this purpose. These two steps are necessary for our ultimate goal to enable inverse biomechanical modelling of the lips, oral cavity and tongue, in order to retrieve patient-specific muscle activation signals inducing oral functions. These activation signals are needed to enable prediction of functional consequences after surgery. Besides patient-specific activation signals, patient-specific anatomical information and tissue parameters are required for simulating treatment effects. The current model does not account for these aspects yet. However, a biomechanical model should incorporate this physical relationship and patient-specific parameters before mimicking performed treatments. The simulated activations controlling the biomechanical model should be similar to the actual muscular control of the patient. sEMG is an instrument to provide the information for these simulated activations. Unfortunately, sEMG is a rough estimate, because of a nonlinear relationship, crosstalk, misplacement of electrodes and other artefacts. We showed that the relationship between 3D lip motion and sEMG can be accurately described by a statistical model. So it can be expected that with our approach the ambiguity problem of inverse modelling can be solved. Our next studies will focus on the relationship between activation signals and sEMG in biomechanical models.

## Conclusion

This study presented a next step towards the personalisation of the functional outcome assessment after treatment of oral cancer. The two dynamic modelling methods proved that a continuum of 3D lip positions can be predicted based on volunteer-specific sEMG features. The discrete Kalman filter with a first-order state estimation performs slightly better than a second-order system, with a mean RMS error of 2.43 mm. The optimal sEMG processing configuration was found to be the WAMP feature with $x_{\lim }$ = 10 mV and a window length of 200 ms. In future studies, this method may be used to solve the problems concerning inverse modelling in biomechanical models, by reduction in the solution space and including patient-specific information.
